# Physical Disability and Its Global Impact on Quality of Life, Activities of Daily Living, and Sleep Quality: A Narrative Review

**DOI:** 10.7759/cureus.95594

**Published:** 2025-10-28

**Authors:** Kiruthika Narayanan, Shanthi Edward, Krishna Prasanth

**Affiliations:** 1 Community Medicine, Sree Balaji Medical College and Hospitals, Chennai, IND; 2 Community Medicine, Sree Balaji Medical College and Hospital, Chennai, IND

**Keywords:** activities of daily living (adl), disabled persons, global public health, physical disability, quality of life (qol), quality of sleep, rehabilitation services

## Abstract

This narrative review explores the global impact of physical disability on quality of life (QOL), activities of daily living (ADL), and sleep quality. Disability encompasses impairments in body function or structure as well as limitations in daily activities and social participation, significantly affecting functional autonomy and well-being. The review highlights how physical disabilities pose challenges to independence and increase reliance on caregiving, with severity varying widely among individuals. It also addresses the critical role of sleep disturbances in exacerbating physical frailty and reducing QOL, particularly in aging populations. The review synthesizes evidence from global, Asian, and Indian contexts, emphasizing the multifaceted nature of disability and the need for comprehensive assessment tools. Gaps in current research, especially regarding integrated analysis of disability, ADL, and sleep quality globally, are identified. The review highlights the need for person-centered strategies and stronger policy frameworks to enhance health outcomes and QOL for people with physical disabilities. It offers a cross-contextual synthesis to inform evidence-based rehabilitation and policies, especially targeting adults with physical disabilities, with special emphasis on older adults given their increased vulnerability to lowered QOL and sleep disturbances, particularly in low- and middle-income countries.

## Introduction and background

The World Health Organization (WHO) conceptualizes health not merely as the absence of illness, but as a holistic state encompassing physical, mental, and social well-being [1]. Physical health refers to the well-being and proper functioning of the body, achieved through regular physical activity, balanced nutrition, and the absence of physical illness or injury [1,2]. Disability represents a universal dimension of human existence, affecting individuals across all socioeconomic and demographic groups [3]. Disability is a physical or mental condition that restricts a person's movements, senses, or activities [2,4]. According to the WHO, it is estimated that around 1.3 billion individuals, constituting nearly 16% of the global population, are currently living with substantial disabilities [3]. The aging population and rising prevalence of disease and disability present major public health challenges, impacting quality of life and increasing demand for medical, social, and long-term care services [4]. Disability encompasses physical impairments, daily activity challenges, and restricted social participation, often reducing independence and increasing reliance on caregivers, making ADL assessment vital for determining functional status and care needs [5]. Sleep disturbances are a major public health concern linked to physical frailty in older adults, as quality sleep is essential for hormone balance, tissue repair, energy regulation, cognitive performance, and cardiovascular health [6]. Previous reviews have mostly studied disability or quality of life separately and focused on high-income countries, with few addressing physical disability alongside functional independence (ADL) and sleep quality across global, regional, and Indian contexts. This review fills that gap by integrating evidence on the interaction of physical disability with QOL, ADL, and sleep, providing essential insights for context-specific rehabilitation and policy planning. The primary objective is to synthesize evidence from global, Asian, and Indian contexts regarding the relationship between physical disability and quality of life (QOL), activities of daily living (ADL), and sleep quality. The study aims to identify key determinants that affect QOL and ADL, with a particular focus on the aging population among physically disabled individuals. Additionally, it seeks to highlight research gaps and discuss policy implications to improve the functional independence and overall well-being of people with physical disabilities.

## Review

Methodology

This study uses a state-of-the-art narrative review design, focusing on recent and relevant evidence from 2000 to 2024 to provide a comprehensive synthesis of trends in disability research. It emphasizes conceptual, epidemiological, and policy aspects instead of quantitative meta-analysis. The narrative synthesis approach enables an in-depth examination of varied literature types, including primary studies, reviews, and policy documents. A thorough literature search was performed in PubMed, Scopus, and Web of Science using MeSH terms and keywords such as “physical disability,” “quality of life,” “WHOQOL,” “activities of daily living,” “Barthel Index,” “sleep quality,” “Pittsburgh Sleep Quality Index,” “rehabilitation,” and “global health.” Additional relevant sources were identified through manual searches of reference lists, WHO reports, national surveys, and other literature. Data saturation was approached through broad database searching combined with manual inclusion till no new themes emerged, ensuring comprehensive coverage of the topic. Two reviewers (KN and SE) independently screened titles and abstracts for eligibility. Full texts of potentially relevant articles were retrieved and assessed independently by both reviewers against the selection/exclusion criteria. Discrepancies were resolved by consensus; unresolved disagreements were adjudicated by a third reviewer (KP). Duplicate records were removed prior to screening. 

Selection Criteria

Peer-reviewed original research, systematic reviews, and narrative reviews addressing physical disability and any of the following outcomes: quality of life (QOL), activities of daily living (ADL), and sleep quality; studies published in English between 2000 and 2024; studies reporting primary data or synthesis relevant to the stated objectives.

Exclusion Criteria

**Figure 1:**
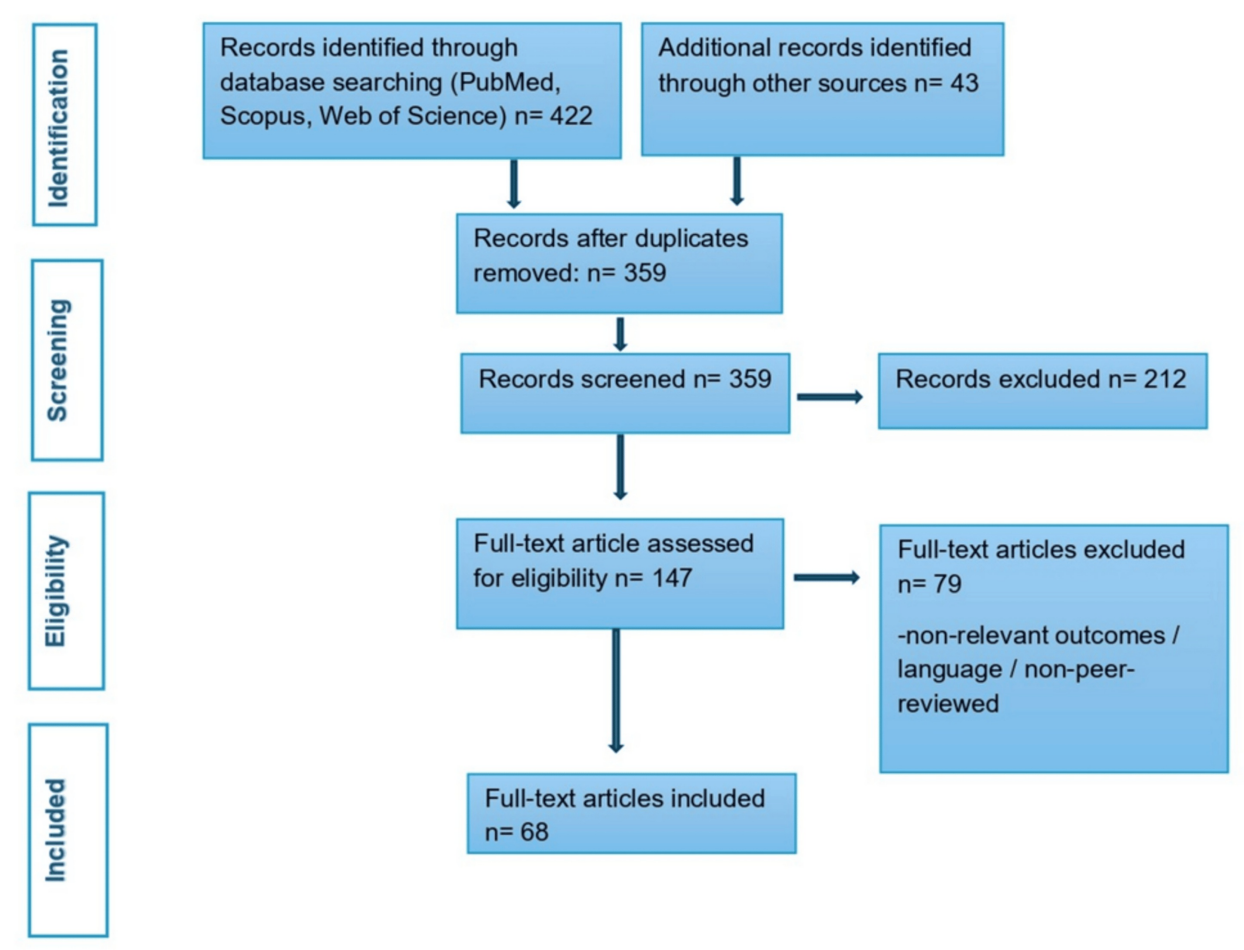
PRISMA-style flow diagram depicting the identification, screening, eligibility, and inclusion of studies. PRISMA, Preferred Reporting Items for Systematic Reviews and Meta-Analyses

Case reports, conference abstracts without full text, non-English articles, and studies focused exclusively on purely cognitive or psychiatric disabilities without a physical disability component, and childhood disabilities. A Preferred Reporting Items for Systematic Reviews and Meta-Analyses (PRISMA)-style diagram summarizing the process is presented in Figure 1.

All 68 articles were quality appraised using the Scale for the Assessment of Narrative Reviews (SANRA). Outcomes were narratively compared across different periods, noting improvements in accessibility mainly in high-income settings, while low- and middle-income countries showed persistent gaps. Measurement differences and evolving disability definitions were accounted for, prioritizing comparable studies to distinguish real trends from methodological changes.

Historical perspective of disability

The concept of disability has evolved considerably across different eras. In early civilizations, disabilities were often associated with spiritual or supernatural explanations, frequently seen as divine punishment [7]. During the 19th and early 20th centuries, the prevailing medical model regarded disability primarily as an individual deficit requiring medical intervention or cure [8]. Toward the late 20th century, a paradigm shift led to the social model of disability, which emphasizes that barriers within society, rather than individual limitations, are the major contributors to disability [9].

The WHO’s International Classification of Functioning, Disability and Health (ICF), introduced in 2001, represents a significant milestone in understanding disability as a complex interaction between health conditions, personal factors, and environmental factors [1]. This biopsychosocial model has fundamentally changed how researchers and practitioners approach disability assessment and intervention.

Definitions and conceptual framework

Disability

According to the WHO, disability refers to an umbrella term encompassing impairments, participation restrictions, and activity limitations [3]. An impairment is defined as a problem in body structure or function; an activity limitation is a difficulty encountered by an individual in executing a task or action; and a participation restriction is a problem experienced by an individual in involvement in life situations [2].

The United Nations Convention on the Rights of Persons with Disabilities (UNCRPD) defines persons with disabilities as "those who have long-term physical, mental, intellectual or sensory impairments which in interaction with various barriers may hinder their full and effective participation in society on an equal basis with others" [10].

Physical Disability

Physical disabilities include impairments in movement, endurance, or physical functioning. Numerous problems are included in it, such as neurological disorders, cardiovascular diseases, musculoskeletal disorders, and congenital abnormalities that impact physical function [11]. International Classification of Diseases (ICD-11) provides a detailed categorization of physical impairments and their functional implications [12]. The most commonly used classification systems include etiology-based, anatomical, and functional classifications.

Etiological Classification

Congenital disabilities are present at birth due to genetic, developmental, or birth-related factors and often require lifelong management. Acquired disabilities develop after birth from injuries, diseases, or degenerative conditions such as spinal cord or brain injuries, amputations, and progressive disorders [13].

Anatomical Classification

Neurological disabilities affect the nervous system, musculoskeletal disabilities impair movement and structure, and sensory-motor disabilities combine sensory and motor impairments [14].

Functional Classification

The International Classification of Functioning, Disability and Health offers a complete framework that links body functions, activities, participation, and environmental factors to assess disability [15].

Table 1 categorizes physical disabilities based on etiological, anatomical, and functional perspectives. It highlights key examples under each classification and outlines the associated functional limitations, emphasizing the multidimensional nature of disability as defined by the WHO’s International Classification of Functioning, Disability and Health (ICF) framework.

**Table 1:** Classification of physical disabilities ICF, International Classification of Functioning, Disability, and Health; ADL, activities of daily living

Type	Examples	Functional impact
Etiological (congenital/acquired) [13]	Spina bifida, cerebral palsy, spinal cord injury, stroke	Mobility, self-care, endurance limitations
Anatomical (neurological, musculoskeletal, sensory-motor) [14]	Arthritis, amputations, neuropathies	Impairments in movement, coordination
Functional (ICF framework) [15]	Participation restrictions, activity limitations	Social participation, ADL deficits

Table 2 presents the principal standardized instruments used to assess QOL, ADL, and sleep quality among individuals with physical disabilities. It summarizes each tool’s domain, purpose, and common research applications.

**Table 2:** Major assessment tools used in research. QOL, quality of life; WHOQOL-BREF, World Health Organization Quality of Life-Brief Version; ADL, activities of daily living; PSQI, Pittsburgh Sleep Quality Index

Domain	Assessment Tool	Description	Common use
QOL	WHOQOL-BREF [16]	Measures multiple domains of quality-of-life	Population-based surveys, clinical research
ADL	Barthel Index [5]	Assesses self-care and independence	Rehabilitation, disability studies
Sleep	PSQI [17]	Evaluates subjective and objective sleep quality	Clinical sleep research

Quality of Life

According to the WHO, QOL is a subjective, multidimensional concept reflecting an individual’s perception of their well-being within their cultural and value context, influenced by physical, psychological, social, and environmental factors. [18].

Activities of Daily Living

ADL represents basic self-care tasks like bathing, dressing, toileting, transferring, and feeding. Instrumental ADLs (IADL) include more complex skills such as managing finances, transportation, and household tasks. The ability to perform ADLs independently is considered a fundamental indicator of functional status and is closely linked to QOL and overall well-being. For individuals with physical disabilities, ADL assessment provides crucial information about their level of independence and care needs [5].

Sleep Quality

Sleep quality is a multidimensional construct encompassing quantitative aspects (sleep duration, sleep latency, number of awakenings) and subjective aspects (depth of sleep, restfulness upon awakening, and overall sleep satisfaction). The Pittsburgh Sleep Quality Index (PSQI), developed by Buysse et al., remains the gold standard for assessing subjective sleep quality [17].

**Figure 2:**
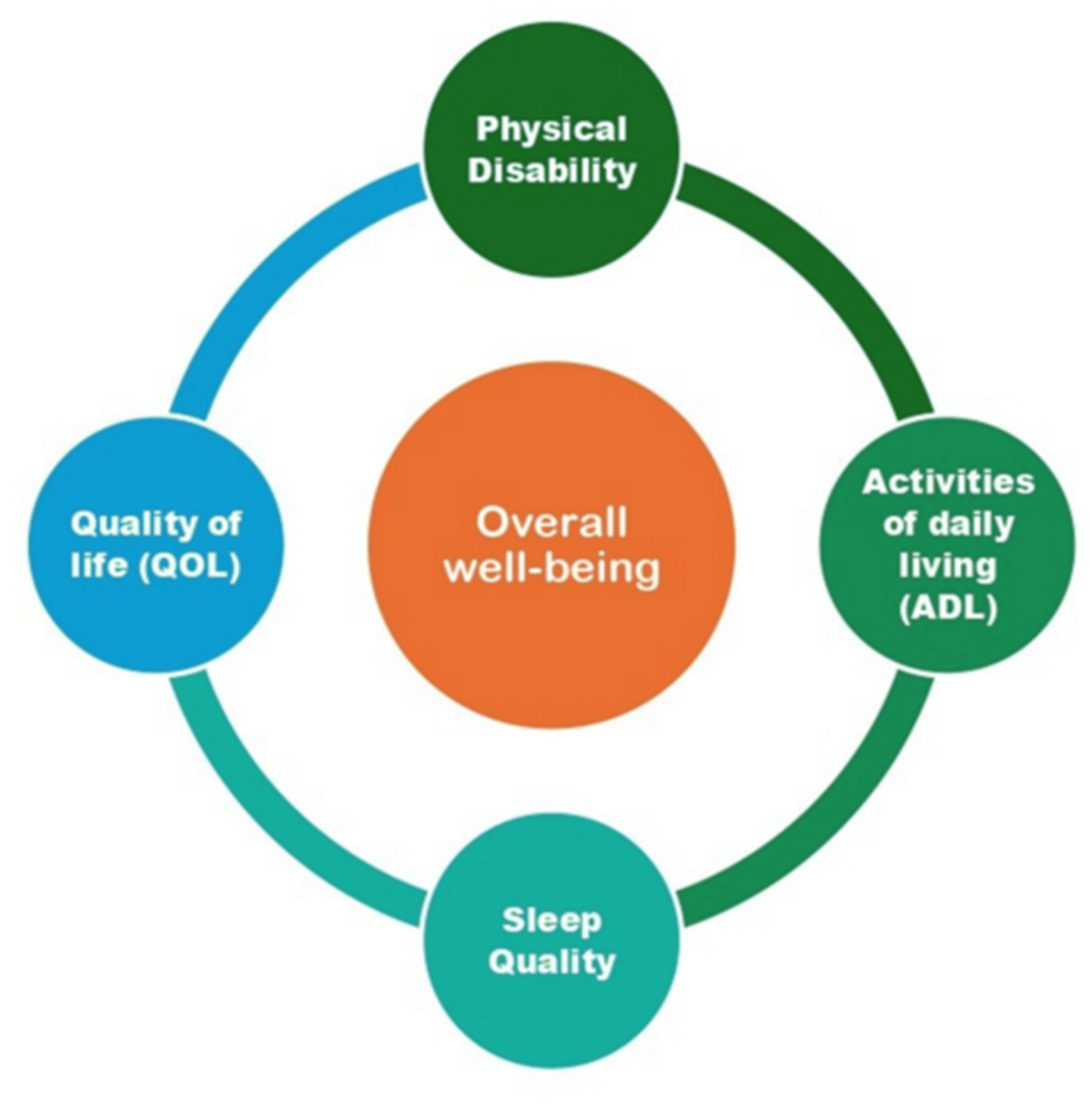
Conceptual framework illustrating the relationship between disability, QOL, ADL, sleep quality, and overall well-being The image is Dr. Kiruthika Narayanan's original creation, developed specifically for this research work. It has not been reproduced, reprinted, or adapted from any previously published source. QOL, quality of life; ADL, activities of daily living

Sleep disturbances are increasingly recognized as significant health issues among individuals with disabilities, affecting their physical health, cognitive function, and overall QOL. The bidirectional relationship between disability and sleep problems creates a complex interplay that requires comprehensive assessment and intervention (Figure 2) [19].

Global scenario of disability

Epidemiological Overview

Recent global estimates indicate that approximately 1.3 billion people, roughly 16% of the world's population-live with significant disability [3]. Physical disabilities constitute a substantial proportion of the global disability burden [5]. The Global Burden of Disease Study 2019 projected that musculoskeletal disorders alone affect over 1.7 billion people globally, making them the leading cause of disability worldwide [20]. Neurological disorders, including stroke, spinal cord injuries, and multiple sclerosis, contribute significantly to the physical disability burden [14]. Regional variations in disability prevalence reflect differences in healthcare access, environmental factors, and demographic patterns. Low- and middle-income countries often experience higher disability prevalence due to factors including inadequate healthcare infrastructure, occupational hazards, and limited preventive services [21].

*QOL* *in Global Context*

Research from throughout the world has repeatedly shown that people with physical impairments have a worse QOL than people in general [4,18]. A systematic review by Tough et al. found that adults with physical disabilities reported significantly lower scores across all domains of QOL, including physical health, psychological well-being, social relationships, and environmental factors [15].

The European Quality of Life Survey revealed significant disparities in QOL between disabled and non-disabled populations across European Union countries, with disabled individuals reporting lower satisfaction with life, health, and social relationships. Similar patterns have been observed in North American populations, where the Centers for Disease Control and Prevention's Behavioral Risk Factor Surveillance System data show consistent QOL disparities [22].

Global Patterns in ADL Limitations

The WHO SAGE study shows that individuals with severe physical impairments are three to five times more likely to experience difficulties in basic self-care activities, making physical disability the strongest predictor of ADL limitations [23].

Cross-cultural studies reveal that variations in ADL performance among physically disabled individuals are influenced by healthcare access, social support, and cultural attitudes, with developed countries generally exhibiting better outcomes due to advanced healthcare and assistive technologies [24].

Sleep Quality: Global Perspectives

International research, including a multinational study by Hirshkowitz et al., has found that adults with physical disabilities experience sleep disorders at rates two to three times higher than the general population, highlighting the significant prevalence of sleep disturbances in this group [17].

The International Classification of Sleep Disorders recognizes several sleep-related issues specifically associated with physical disabilities, including sleep-related movement disorders, sleep-related breathing disorders secondary to neuromuscular conditions, and circadian rhythm sleep-wake disorders [19].

While global evidence highlights common trends, region-specific variations necessitate separate examination, beginning with Asia.

Asian scenario

Disability Prevalence and Characteristics

Asia has the greatest number of people with disabilities worldwide since it is home to over 60% of the world's population [3]. The Asia-Pacific area is home to more than 690 million people with disabilities, according to the Asian and Pacific Decade of Disabled Persons (2013-2022) [25]. Disability prevalence in the Asia-Pacific varies widely due to differences in definitions and data methods, ranging from 1.5% in Cambodia to 18.5% in New Zealand, with physical impairments accounting for 40%-50% of disabilities in the region according to Economic and Social Commission for Asia and the Pacific (ESCAP) [26].

QOL in Asia

Asian research, particularly from Japan, indicates that social participation and community integration are stronger predictors of QOL for physically disabled individuals than the severity of their physical impairments [27]. Studies from South Korea have demonstrated that family support and social acceptance significantly influence QOL outcomes for physically disabled persons, reflecting the importance of collective cultural values in Asian societies [28]. Chinese research has emphasized the role of traditional medicine and holistic approaches in improving QOL for individuals with physical disabilities [29].

ADL Pattern in Asian Populations

Research, including a multi-country study by Ng et al., highlights that family caregiving patterns and intergenerational support significantly influence independence in ADL among physically disabled individuals in Asian populations [30]. Japanese longitudinal studies have shown that early rehabilitation and adaptive equipment provision are crucial factors in maintaining ADL independence among individuals with acquired physical disabilities [31]. Thai research has highlighted the importance of culturally appropriate ADL assessments that consider local customs and practices [32].

Sleep Quality Research in Asia

Asian research from Singapore shows that environmental factors like noise pollution and poor housing conditions significantly affect sleep quality among urban physically disabled populations [33]. Korean research has emphasized the role of social stigma and psychological stress in contributing to sleep problems among disabled individuals [34].

Within Asia, India presents a particularly distinctive scenario, shaped by its vast population, socioeconomic disparities, and evolving legislative frameworks, warranting a focused examination of disability, QOL, ADL, and sleep quality in the Indian context.

Indian scenario

Disability Statistics and Demographics

According to the 2011 Census, India has the largest population of people with disabilities globally, totaling 26.8 million individuals, which is 2.21% of the population; however, the 2016 Rights of Persons with Disabilities Act expanded the definition of disability, and recent studies suggest the actual prevalence may be higher [35]. The National Sample Survey Office (NSSO) 76th round survey on persons with disabilities (2018) reported that 2.2% of India's population has impairment of some kind; locomotor disability is the most common type, affecting 20.3 per 1000 population [36]. The survey revealed significant rural-urban disparities, with rural areas having higher disability prevalence rates [37].

Socioeconomic Impact

Physical disability in India is closely linked to poverty and social exclusion, with disabled households having significantly lower per capita income and only about 30% of working-age disabled individuals participating in the labor force [38]. Educational attainment is also much lower, with over 60% lacking formal education, which limits employment opportunities and perpetuates poverty cycles [39].

Legislative Framework and Policy Development

India's approach to disability has evolved significantly over recent decades, with the Rights of Persons with Disabilities Act 2016 replacing the 1995 act and expanding recognized disabilities from seven to twenty-one [35]. The Accessible India Campaign, launched in 2015, aims to create a barrier-free society by ensuring universal accessibility in public buildings, transportation, and information systems, thereby enabling persons with disabilities to live independently and participate fully in all aspects of life [40].

QOL Research in India

Studies in Indian states consistently show that socioeconomic status, education, and healthcare access are major determinants of QOL for physically disabled individuals, while research by Sharma and colleagues highlights social stigma and discrimination as significant negative influences on their QOL [41].

Sleep Quality Research in India

Limited research in India shows a high prevalence of sleep disturbances among physically disabled individuals, with contributing factors including pain, medication effects, environmental conditions, and psychological distress; traditional Ayurvedic sleep management is commonly used alongside conventional treatments, though systematic studies on their combined effectiveness are lacking [42].

National initiatives supporting rehabilitation of persons with disabilities in India

Deendayal Disabled Rehabilitation Scheme

The Deendayal Disabled Rehabilitation Scheme (DDRS), established by the Ministry of Social Justice and Empowerment, provides financial support to NGOs to deliver rehabilitation services for persons with disabilities, including early intervention, education, vocational training, and community-based rehabilitation programs [43].

Sugamya Bharat Abhiyan (Accessible India Campaign)

Launched in 2015, this flagship program aims to create barrier-free environments for persons with disabilities. The campaign focuses on accessibility in public buildings, transportation systems, and information and communication technology [44].

National Program for Rehabilitation of Persons with Disabilities (NPRPD)

The NPRPD provides funding support to state governments and union territories for implementing rehabilitation programs. The program emphasizes community-based rehabilitation and aims to ensure that rehabilitation services reach the grassroots level [45].

District Disability Rehabilitation Center (DDRC) Scheme

This scheme aims to provide comprehensive rehabilitation services at the district level, including identification, assessment, treatment, and rehabilitation for the disabled. The DDRCs se as focal points for disability services in their respective districts [46].

Unique Disability ID (UDID) Project

The goal of the UDID project is to compile a database of people with impairments and provide them with Unique Disability Identification Cards. This initiative facilitates transparent delivery of government schemes and services to disabled individuals [47].

Insights from the Indian context further underscore the complexity of disability-related challenges and highlight the need to synthesize findings across global, regional, and national levels to identify commonalities, gaps, and future directions.

Tamil Nadu scenario: State-level perspectives

Disability Profile of Tamil Nadu

Approximately 1.86 million people with disabilities reside in Tamil Nadu, making up 2.54% of the state's total population, according to the 2011 Census [35]. The state has one of India's highest rates of disability prevalence, with the most prevalent kind being locomotor impairment [48]. The Tamil Nadu State Commissioner for Persons with Disabilities reports that physical disabilities make up about 45% of all disabilities in the state, with Chennai and nearby districts such as Chengalpattu having significant populations of physically disabled individuals due to urbanization and industrial activities [49].

State Policies and Programs

Tamil Nadu has been a pioneer in disability rights and services in India, establishing a separate department for differently-abled welfare as early as 1987; the Tamil Nadu Rights of Persons with Disabilities Rules 2017 provide detailed implementation guidelines for central legislation, and the state runs numerous rehabilitation centers alongside schemes such as disability pensions, assistive devices, and skill development programs, while the Chennai Corporation has implemented multiple accessibility initiatives under the Accessible India Campaign [44,50].

Healthcare Infrastructure and Services

Tamil Nadu has many NGOs working in disability rehabilitation, especially in urban areas like Chennai, which have better healthcare access; the Chengalpattu district, part of the Chennai metropolitan area, benefits from relatively better healthcare facilities compared to other rural districts in the state [51].

Chennai and Chengalpattu District Context

Chennai, as the capital and major metropolitan center, offers relatively better accessibility infrastructure, specialized healthcare services, and employment opportunities for physically disabled individuals compared to other regions, but challenges remain with last-mile connectivity, inconsistent accessibility in public transport and buildings, and ongoing efforts are focused on making the city more inclusive through universal design and accessibility initiatives [52].

Chengalpattu district, characterized by a mix of urban and rural areas, faces broader challenges for disabled individuals but benefits from ongoing development projects aimed at improving accessibility and rehabilitation services, supported by government schemes, NGOs, and coordinated district-level initiatives focusing on healthcare, skill development, and social welfare [53].

Table 3 illustrates a comparative synthesis of Indian and international research exploring how physical disability affects QOL, ADL, and sleep quality. This reveals consistent determinants, advancing age, gender inequities, educational deprivation, socioeconomic disadvantage, marital disruption, and adverse childhood experiences (ACEs), that collectively undermine independence and psychosocial well-being. Studies from diverse settings (India, China, Canada, the United States, and Taiwan) demonstrate convergent evidence linking disability with deteriorating QOL and functional outcomes. These findings advocate for multidisciplinary, context-specific interventions integrating physical rehabilitation, mental health support, and socioeconomic inclusion to enhance the QOL of persons with physical disabilities.

**Table 3:** Comparative summary of global and Indian studies on physical disability, quality of life, activities of daily living, and sleep quality. QOL, quality of life; WHOQOL-BREF, World Health Organization Quality of Life-Brief Version; ADL, activities of daily living; OR, odds ratio; CI, confidence interval; AOR, adjusted odds ratio

Domain	Study	Key findings	Comparative insight
Age and Disability Correlation	Hazra et al., 2023 (Kolkata, India) [54]	Among 57.9% elderly participants, younger adults demonstrated superior QoL owing to fewer comorbidities and enhanced coping capacity.	Younger adults retain better functional independence; aging inversely affects QOL.
	Kuvalekar et al., 2015 (Udupi, India) [55]	Older adults had significantly reduced QOL across all WHOQOL-BREF domains.	Reinforces negative correlation between increasing age and QOL among disabled individuals.
	Ramadass et al., 2018 (Rural North India) [56]	Age ≥ 60 years associated with reduced QOL and increased disability (AOR = 12.3; 95% CI 4.45-33.97; *P* < 0.001).	Aging acts as a critical determinant of dependency and functional decline.
	Medhi et al., 2021 (Northeast India) [57]	Functional disability more prevalent in advanced age groups (OR = 5.47; 95% CI 2.62-11.42; *P* < 0.01).	Findings consistent across regions; aging linked with diminished ADL independence.
Gender Differences	Khan et al., 2018 (Haryana, India) [58]	Females had higher disability risk (OR = 2.0; *P* < 0.05).	Contrasts with current study where males showed higher ADL dependence, possibly due to occupational and behavioral factors.
Educational Status	Medhi et al., 2021 (Northeast India) [57]	Illiteracy correlated with greater functional disability (OR = 2.2; *P* = 0.05).	Education improves adaptive capacity and enhances QOL in disabled populations.
	Kumar et al., 2014 (Puducherry, India) [59]	Lower educational attainment predicted poorer QOL (*P* = 0.004).	Supports educational empowerment as a pivotal factor for disability rehabilitation.
Socioeconomic Status (SES)	Qiu et al., 2023 (China) [60]	Disability likelihood increased in low-SES groups (OR = 0.679; *P* ≤ 0.05). Confirms global pattern of economic inequity aggravating disability outcomes.	
Adverse Childhood Experiences (ACE)	Li et al., 2022 (China, CHARLS Cohort) [61]	≥ 4 ACEs associated with higher odds of ADL disability (OR = 1.32-1.41); chronic diseases mediated > 60% of this relationship.	Early-life adversity predisposes to persistent functional limitations and reduced QOL in later life.
Marital Status	Savage and McConnell, 2016 (Canada) [62]	Disabled women more likely to be unmarried, impacting QOL (OR = 1.37; 95% CI 1.29-1.14).	Marital instability contributes to social isolation and ADL dependency.
Sleep Quality	Chien and Chen, 2015 (Taiwan) [63]	Individuals with disabilities had higher mean PSQI scores (8.3 ± 3.9 vs. 6.9 ± 4.3; *P* < 0.004).	Sleep disturbance intensifies physical and psychological distress in disabled adults.
	Brightman et al., 2024 (the United States) [64]	Disability independently predicted poor sleep quality and reduced efficiency.	Highlights sleep impairment as a critical but under-addressed component of disability management.

Despite extensive global evidence, there remains a paucity of region-specific research addressing the interrelationship between QOL, ADL, and sleep quality among physically disabled individuals within these districts. This gap highlights the need for localized, context-specific studies to capture regional determinants of disability and guide targeted interventions and inclusive policy measures aimed at improving independence and overall well-being. The global impact of physical disability is inherently multidimensional, encompassing not only biomedical consequences but also social, psychological, and structural determinants that collectively shape individual well-being. Recognizing this complexity aligns with the objectives of the present review, which integrates evidence across QOL, functional independence, and sleep health. Such a multidimensional synthesis offers comprehensive insight into the interlinked challenges faced by persons with disabilities and serves as a foundation for context-sensitive policy formulation and targeted public health interventions.

Future directions and recommendations

Several important questions remain unanswered, particularly concerning the dynamic interplay between physical disability and evolving social roles, economic dependence, and health systems over time. Future research should prioritize longitudinal and mixed-methods designs to capture changes in QOL, ADL, and sleep across different life stages. Emphasis on gender-specific differences, digital rehabilitation technologies, and integration of psychosocial support within care models will strengthen the evidence base. Developing standardized, culturally adaptable measurement tools for low- and middle-income contexts is essential. Policymakers should focus on inclusive rehabilitation, community-based assistive technology, and longitudinal monitoring systems to translate research into effective interventions that enhance independence and well-being for physically disabled populations. These directions will pave the way for targeted, evidence-based improvements in care and policy, addressing current knowledge gaps and unmet needs.

## Conclusions

This narrative review underscores the profound and multidimensional impact of physical disability on quality of life, activities of daily living, and sleep quality across global, Asian, and Indian contexts. The evidence reveals that disability is not confined to functional impairments but extends to social participation, independence, psychological well-being, and overall health outcomes. While high-income countries have advanced healthcare systems and support structures that mitigate some of these challenges, individuals in low- and middle-income nations, including India, continue to experience compounded difficulties due to poverty, stigma, inadequate rehabilitation, and limited policy implementation. Sleep disturbances, often overlooked, emerged as a significant contributor to physical frailty and diminished quality of life, highlighting the necessity of integrating sleep health into disability care. Additionally, assessment of activities of daily living remains critical for determining functional autonomy and tailoring rehabilitation strategies. Despite existing initiatives and policies in India, research gaps remain evident, particularly in linking disability with sleep health and long-term quality of life outcomes. A lack of comprehensive analytical studies and culturally sensitive assessment tools continues to hinder effective intervention planning. Future directions must focus on multidisciplinary, person-centered strategies that integrate medical care, psychosocial support, and policy reforms. Strengthening rehabilitation frameworks, fostering community-based support systems, and ensuring accessibility in healthcare and infrastructure are essential to improving the overall well-being of individuals with physical disabilities. Addressing these challenges through research, advocacy, and inclusive policymaking can contribute substantially to achieving equity, empowerment, and enhanced quality of life for this vulnerable population.
